# Iodine in Snake Bite

**Published:** 1851-01

**Authors:** 


### ARTICLE XI.

*Snake Bite cured by the Tinc. of Iodine.*—Dr. James Langworthy of Reynoldsburg, has kindly forwarded the following observation:

C. G., a boy about 8 years old, was bitten by a snake about 2 o'clock P. M., August 28th, 1850. I was called in about 5 o'clock the same day. Found a characteristic wound on calf of the leg. The limb had swollen very much; was quite tender, but there was no discoloration, except very near the wound. The usual applications recommended in such cases were used, and continued until the next morning, but to no effect. The swelling increased, and by that time had extended over the whole limb, the inflammation becoming erysipalatous and of an aggrivated character.

Recollecting a notice of the use of Tinc. Iodine in such cases, in Braithwaite's Retrospect, part 20, pp, 180. [Dr. Whitmire's article first published in the North Western Medical and Surgical Journal.] I applied it three times on the more inflamed parts, and twice upon the whole leg at intervals of twelve hours with the most gratifying success. It seemed to act like a charm, and completely subdued the inflammation. —*Ohio Med. Jour.*

